# Using a decline in serum hCG between days 0–4 to predict ectopic pregnancy treatment success after single-dose methotrexate: a retrospective cohort study

**DOI:** 10.1186/1471-2393-13-30

**Published:** 2013-02-01

**Authors:** Monika Skubisz, Philip Dutton, William Colin Duncan, Andrew W Horne, Stephen Tong

**Affiliations:** 1 Translational Obstetrics Group, University of Melbourne, Mercy Hospital for Women, Heidelberg, Victoria 3084, Australia; 2 The Ritchie Centre, Monash Institute of Medical Research, Monash University, Clayton, Victoria 3168, Australia; 3 MRC Centre for Reproductive Health, University of Edinburgh, Edinburgh, EH16 4TJ, United Kingdom

**Keywords:** Ectopic pregnancy, Human chorionic gonadotrophin, Medical management, Methotrexate, Positive predictive value, Treatment success

## Abstract

**Background:**

The current measure of treatment efficacy of single-dose methotrexate for ectopic pregnancy, is a fall in serum hCG of ≥15% between days 4–7 of treatment, which has a positive predictive value of 93% for treatment success. Two small studies have proposed a fall in serum hCG between days 0–4 after treatment confers similar, earlier prognostic information, with positive predictive values of 100% and 88% for treatment success. We sought to validate this in a large, independent cohort because of the potentially significant clinical implications.

**Methods:**

We conducted a retrospective study of women (n=206) treated with single-dose methotrexate for ectopic pregnancy (pre-treatment serum hCG levels ≤3000 IU/L) at Scottish hospitals between 2006–2011. Women were divided into two cohorts based on whether their serum hCG levels rose or fell between days 0–4 after methotrexate. Treatment outcomes of women in each cohort were compared, and the test performance characteristics calculated. This methodology was repeated for the current measure (≥15% fall in serum hCG between days 4–7 of treatment) and an alternate early measure (<20% fall in serum hCG between days 0–4 of treatment), and all three measures were compared for their ability to predict medical treatment success.

**Results:**

In our cohort, the positive predictive value of the current clinical measure was 89% (95% CI 84-94%) (121/136). A falling serum hCG between days 0–4 predicted treatment success in 85% (95% CI 79-92%) of cases (94/110) and a <20% fall in serum hCG between days 0–4 predicted treatment success in 94% (95% CI 88-100%) of cases (59/63). There was no significant difference in the ability of these tests to predict medical treatment success.

**Conclusions:**

We have verified that a decline in serum hCG between days 0–4 after methotrexate treatment for ectopic pregnancies, with pre-treatment serum hCG levels ≤3000 IU/L, provides an early indication of likelihood of treatment success, and performs just as well as the existing measure, which only provides prognostic information on day 7.

## Background

Ectopic pregnancies occur in 1-2% of pregnancies [1]. Although potentially life threatening, the ability to non-invasively detect ectopic pregnancies before they rupture with ultrasound affords some women the option of medical management. Stovall *et al.*[2] first demonstrated the safety and efficacy of outpatient methotrexate to treat women with ectopic pregnancies in 1989, and today, approximately 25-30% of women presenting with this condition are eligible for such treatment [3,4].

Quantification of serum hCG provides a sensitive biomarker of viable trophoblastic tissue and is used in the medical treatment of ectopic pregnancy to monitor treatment response. In the single-dose methotrexate protocol developed by Stovall *et al.*[2,5], treatment efficacy is determined by a ≥15% fall in serum hCG between days 4 and 7 of treatment; if there has been an insufficient fall in serum hCG at this time, further doses of methotrexate and/or surgery are indicated. This measure has been validated by Kirk *et al.*[6] and shown to have a positive predictive value of 93% for treatment success. By definition, however, the first indication of treatment efficacy can be ascertained no earlier than day 7.

In 2010, Nguyen *et al.*[7] reported that a fall in serum hCG between days 0–4 after methotrexate injection predicted treatment success with no further intervention in, remarkably, 100% of cases (n=30). Investigating a cohort of 45 women in a follow-up study, we reported that a fall in serum hCG between days 0–4 predicted treatment success after single-dose methotrexate in 88% of cases [8]. Furthermore, an earlier study by Agostini *et al.*[9] of 129 cases of ectopic pregnancy reported that a ≥20% fall in serum hCG between days 1–4 after medical treatment with a single dose methotrexate had a positive predictive value of 97% for treatment success.

The fact that serum hCG trends may be able to accurately predict treatment outcomes for many women as early as day 4 has potentially important clinical implications. Firstly, it significantly reduces the duration of anxious uncertainty patients (and clinical staff) must endure before obtaining an indication of whether or not the treatment is working. Secondly, it raises the possibility of changing protocols in medical management of ectopic pregnancy and intervening with a second dose of methotrexate earlier in cases where serum hCG levels have not fallen between days 0–4, to potentially improve overall methotrexate treatment success rates and/or decrease the length of time required to achieve a successful resolution of the ectopic pregnancy.

However, before proposing a fall in day 0–4 serum hCG should be used clinically as an early prognostic indicator for medical treatment success in management of ectopic pregnancies, we felt it important to perform a further validation using a large, independent cohort, especially given that the two previous studies featured small numbers of women (n=30 and 45, respectively) [7,8]. Therefore, we examined the ability of a fall in serum hCG between days 0–4 to predict treatment success after single-dose methotrexate treatment for ectopic pregnancy in a cohort of 206 Scottish women.

## Methods

### Objective

A retrospective cohort study was performed to assess the prognostic value of a fall in serum hCG between days 0–4 after medical treatment of ectopic pregnancy with single-dose methotrexate.

### Participants

The South East Scotland Research Ethics Service deemed that this study did not require formal NHS ethical review, as the project was audit based and used only data obtained as part of routine care.

Data was collected from electronic and linked records for women treated with single-dose methotrexate for ectopic pregnancy in early pregnancy units in Scotland between 2006 and 2011. Data for the year 2006 was available for women treated at all 11 Scottish early pregnancy units (n=210) and for treatment episodes between 2007 and 2011, records were collected from the Royal Infirmary of Edinburgh’s Pregnancy Support Centre (n=187). Data collected included baseline demographics, treatment dates and outcomes and serial serum hCG measurements.

Available patient data was classified and only included if it fulfilled the ectopic pregnancy diagnostic criteria published in the consensus statement of early pregnancy outcomes [10].

Scottish protocols stipulate that to be eligible for outpatient medical management of ectopic pregnancy, a woman must be haemodynamically stable and reliable for follow up, the pre-treatment serum hCG should be <3000IU/L and the diagnostic ultrasound should show a gestational sac size no greater than 4cm in largest diameter, with little or no pelvic free fluid. Eligible women were treated as outpatients with a single dose of intramuscular methotrexate at 50 mg/m^2^.

Furthermore, to be included in our analysis, participants needed to have a recorded serum hCG on day 0 or 1, day 4 and day 7 of treatment, as well as a documented treatment outcome i.e. successful medical management with or without further doses of methotrexate, or failed medical management requiring surgery.

We have previously noted confusion both clinically and in the literature relating to the day of treatment, with both day 0 and day 1 being used to denote the day of methotrexate injection [8]. The original protocol proposed by Stovall *et al.*[5] considers the day of treatment to be day 1, however, the study by Nguyen *et al.*[7] designated day 0 as the day of treatment. We have thus included women with either a day 0 or day 1 serum hCG recorded.

### Description of analysis

In order to achieve a dichotomous outcome (i.e. success/failure), we have for the purposes of this analysis defined treatment success as a complete resolution of serum hCG to <15IU/L after a single dose of methotrexate with no further intervention, medical or surgical. Thus, cases requiring repeat doses of methotrexate or surgical management were classified under treatment failure in this study.

Participants were divided into two cohorts based on whether their serum hCG rose or fell between days 0–4 after single-dose methotrexate for ectopic pregnancy (day 0/1 serum hCG - day 4 serum hCG). The positive predictive value, negative predictive value, sensitivity and specificity were calculated.

We used this method to assess the prognostic value of other measures of treatment efficacy: 1) a ≥15% fall in serum hCG between days 4–7 of medical treatment ((day 4 – day 7 serum hCG/ day 4 serum hCG) x 100) and 2) a ≥20% fall in serum hCG between days 0–4 of treatment ((day0/1 - day 4 serum hCG/day 0/1 serum hCG) x 100), as proposed by Agostini *et al.*[9].

### Statistical analysis

The ability of each measure to predict single-dose methotrexate treatment success in ectopic pregnancy was compared using Fisher’s exact test. Baseline clinical demographics were compared using a Student’s *t* test for continuous variables and a Fisher’s exact test for categorical variables. For each measure, we calculated sensitivities, specificities, positive and negative predictive values. Statistical analysis was performed using PRISM (GraphPad, La Jolla, CA, USA).

## Results

### Participants

Records were available for a total of 397 women treated with single-dose methotrexate for ectopic pregnancy between 2006–2011, using local protocols based on the RCOG guideline [11]. Of these: 2 were lost to follow up or had no recorded serum hCG levels, 38 had no recorded day 0 or 1 serum hCG, 104 women had no recorded day 4 serum hCG and 32 had no recorded day 7 serum hCG. A further 13 women were excluded for having a pre-treatment serum hCG of <3000IU/L and 2 had no documented treatment outcome. This left 206 women suitable for analysis.

The baseline characteristics of the cohort include a mean day 0 (or day 1) hCG level of 778.2 IU/L (SEM ± 49.25), a mean age of 30.8 (SEM ± 0.4) years, a previous ectopic pregnancy rate of 14% (28/206), previous recorded chlamydial infection in 18% (36/206) and 24% of the cohort were current or past smokers 49/206). All participants demonstrated a gestational sac size of <4cm and up to moderate amounts of pelvic free fluid on ultrasound.

### Treatment outcomes

The treatment success rate achieved with a single dose of methotrexate i.e. without need for a subsequent dose of methotrexate and/or surgery in the overall cohort was 71% (147/206). 40 women (19%) required further doses, so that the overall medical treatment success rate (allowing for additional doses of methotrexate) was 90% (186/206). 20 women (10%) required surgical management.

### Falling hCG between days 0–4 to predict medical treatment success

110/206 women demonstrated a falling serum hCG between days 0–4 of treatment (mean hCG fall −161 IU/L (SEM ± 21.5)); of these, 94/110 women experienced treatment success with a single dose of methotrexate (no repeat doses), giving this measure a positive predictive value of 85% (95% CI 79-92%). The sensitivity and specificity for a falling hCG between days 0–4 to predict single-dose methotrexate treatment success were 64% and 73%, respectively, and the negative predictive value 46%.

Of the remaining 96/206 women who had a rising serum hCG between days 0–4 (mean hCG rise 317 IU/L (SEM ± 32.2)), 53/96 (55%) experienced medical treatment success (i.e. negative predictive value of 45%).

We examined the pre-treatment serum hCG trends of participants with a falling serum hCG between days 0–4, to show that the early falling hCG levels were an effect of treatment and not just a pre-existing trend. The pre-treatment serum hCG trend was available for 98/110 of women with a falling serum hCG betweens days 0–4. Observation of pre-treatment hCG levels ranged from 1–32 days prior to methotrexate administration. A rising hCG trend was noted in 61/98 (62%) women in this cohort prior to treatment, and of these, 52/61 (85%) were successfully treated with one dose of methotrexate; 9/61 (15%) required either additional doses of methotrexate or surgical management. A falling pre-treatment hCG trend was noted in 37/98 women in this cohort, with 31/37 (84%) of these successfully treated and 6/37 (16%) requiring additional doses of methotrexate and/or surgery.

### ≥15% Fall in hCG between days 4–7 to predict medical treatment success

Using the current test of medical treatment efficacy in ectopic pregnancy, 136/206 women demonstrated a ≥15% fall in serum hCG between days 4–7. Of these, 121/136 women experienced treatment success resulting in a positive predictive value of 89% (95% CI 84-94%). Of the 70/206 women whose serum hCG did not fall by ≥15% between days 4–7, 25/70 (36%) experienced treatment success (negative predictive value of 64%). The sensitivity of this measure to predict treatment success was 82% and specificity 75%.

###  ≥20% Fall in hCG between days 0–4 to predict medical treatment success

63/206 women had a ≥20% fall in their serum hCG concentrations between days 0–4. Of these, 59/63 experienced treatment success providing a positive predictive value of 94% (95% CI 88-100%). Of the 143/206 women where serum hCG did not fall ≥20% between days 0–4, 87/143 (61%) experienced treatment success (i.e. negative predictive value 39%). The sensitivity of this measure to accurately predict medical treatment success was 40% and the specificity 93%. The test characteristics of all 3 measures are summarised in Table 1.

**Table 1:** Summary test characteristics of each of the 3 measures analysed

**TEST 1: Falling hCG between days 0-4**					
	**Treatment Success**	**Treatment Failure**	**Total**		
Falling hCG	94	16	110	**PPV:**	85% (95% CI 79-92%)
Rising hCG	52	44	96	**NPV:**	46%
Total	146	60	206		
	**Sensitivity:**	**Specificity:**			
	64%	73%			
**TEST 2: ≥15% Fall in hCG between days 4-7**					
	**Treatment Success**	**Treatment Failure**	**Total**		
Falling hCG	121	15	136	**PPV:**	89% (95% CI 84-94%)
Rising hCG	25	45	70	**NPV:**	64%
Total	146	60	206		
	**Sensitivity:**	**Specificity:**			
	83%	75%			
**TEST 3: <20% Fall in hCG between days 0-4**					
	**Treatment Success**	**Treatment Failure**	**Total**		
Falling hCG	59	4	63	**PPV:**	94% (95% CI 88-100%)
Rising hCG	87	56	143	**NPV:**	39%
Total	146	60	206		
	**Sensitivity:**	**Specificity:**			
	40%	93%			

### Comparison of the predictive value of the 3 measures

There was no significant difference between the ability of any of the tests to accurately predict medical treatment success with a single dose of methotrexate when comparing them individually using Fisher’s exact test (p=≥0.13).

## Discussion

This study shows that a fall in serum hCG between days 0–4 of treatment represents an 85% likelihood of treatment success with no further intervention, medical or surgical, for single-dose methotrexate treatment of ectopic pregnancy. We analysed the early serum hCG trends of 206 women treated with single-dose methotrexate for their ectopic pregnancies in a range of treatment centres, and these are numbers far greater than previously reported (n=30, n=45 and n=129) [7-9].

A large number of women were excluded from our analysis due to inaccurate timing of (or missed) serum hCG level measurements, in contrast to the requirements of the single-dose methotrexate treatment protocol. Specifically, the analyses performed in this study required serum hCG levels to be available for days 0 or 1, day 4 and day 7 after methotrexate treatment. While this raises the possibility of a selection bias, it is not immediately clear how this may have affected our results. Certainly attendance at such numerous and specific time points for serum sampling requires significant patient (and physician) compliance, but non-compliance is unlikely to have any bearing on treatment outcome, which was the primary outcome assessed in this study. Indeed, the results obtained are consistent with those of previous studies in other populations [7-9]. This study therefore, strengthens the validity of the prognostic value of a falling serum hCG between days 0–4 after single-dose methotrexate treatment for ectopic pregnancy.

Treatment success for the purposes of this study was defined more strictly than in the current protocol and clinical practice, in that it did not allow for any additional doses of methotrexate. This was for the purposes of rigorously testing the prognostic value of a falling serum hCG between days 0–4 for an actual single (rather than a variable) dose methotrexate treatment course. Allowing for additional doses of methotrexate is likely to only improve the positive predictive value of this measure.

The application of a quantified ≥20% fall in serum hCG between days 0–4 of medical treatment improves the positive predictive value from 85% to 94%. This is likely explained by the fact that the greater the fall in serum hCG between days 0–4 of treatment, the more likely the patient is to experience treatment success. There is a clear trade-off, however, between increasing accuracy of prediction with such a cut-off (specificity) and clinical applicability of this measure to a greater number of women (sensitivity). The sensitivity of a falling serum hCG between days 0–4 fell substantially from 64% to 40% when a ≥20% cut-off was applied, so that the test could only provide meaningful prognostic information to 29% (59/206) of women. In contrast, prognostic information was available for 53% (110/206) of women if *any* fall in serum hCG between days 0–4 was used as the cut-off for a measure of treatment efficacy, with no difference in prognostic accuracy.

It is possible that women with a falling serum hCG between days 0–4 after single-dose methotrexate for ectopic pregnancies may have had already failing pregnancies and did not require treatment. This was not the case, however, as inspection of the pre-treatment serum hCG trends for women with early falling serum hCG levels after methotrexate indicated that the majority (62%) in fact had rising serum hCG levels prior to treatment. Interestingly, regardless of a rising or falling pre-treatment hCG trend, a falling serum hCG level between days 0–4 predicted single-dose methotrexate treatment success with equal measure in this sub-cohort of women (85% and 84%, respectively).

This study examined ectopic pregnancies with pre-treatment serum hCG levels of ≤3000 IU/L, and the results may not apply to medically treated ectopic pregnancies with pre-treatment serum hCG levels <3000 IU/L. Furthermore, given that a fall in serum hCG between days 0–4 is not 100% predictive of treatment success, it is still prudent to continue monitoring hCG levels until normalisation.

## Conclusions

We have found that a fall in serum hCG between days 0–4 after single-dose methotrexate treatment of ectopic pregnancy (where the pre-treatment serum hCG is ≤3000 IU/L), predicts treatment success in 85% of cases, with no further intervention required (medical or surgical).

With this measure, prognostic information is obtained three days earlier than from the current, standard clinical measure, and with comparable accuracy. This raises the possibility of altering existing methotrexate protocols, with the aim of increasing medical treatment success rates, by intervening with an earlier repeat dose where early serum hCG levels are still rising; this would, however, require further investigation.

We believe that clinical staff caring for women with ectopic pregnancies, treated with single-dose methotrexate, can confidently use a falling serum hCG between days 0–4 of treatment to provide earlier prognostic information and reassurance to patients with this potentially life-threatening condition.

## Competing interests

The authors declare that they have no competing interests.

## Authors’ contributions

ST, AH, CD, MS and PD conceived the study. PD collected the data. ST and MS performed the data analysis and drafted the manuscript. All authors critically reviewed the manuscript and approved the final version.

## Pre-publication history

The pre-publication history for this paper can be accessed here:

http://www.biomedcentral.com/1471-2393/13/30/prepub
